# MiR-142-3p is downregulated in aggressive p53 mutant mouse models of pancreatic ductal adenocarcinoma by hypermethylation of its locus

**DOI:** 10.1038/s41419-018-0628-4

**Published:** 2018-05-29

**Authors:** Jack D. Godfrey, Jennifer P. Morton, Ania Wilczynska, Owen J. Sansom, Martin D. Bushell

**Affiliations:** 1 Medical Research Council Toxicology Unit, Lancaster Rd, Leicester, LE1 7HB UK; 2 Cancer Research UK Beatson Institute, Garscube Estate, Switchback Road, Glasgow, G61 1BD UK; 3 Institute of Cancer Sciences, University of Glasgow, Garscube Estate, Switchback Road Glasgow, Glasgow, G61 1QH UK

## Abstract

Pancreatic ductal adenocarcinoma (PDAC) is an extremely aggressive disease with poor prognostic implications. This is partly due to a large proportion of PDACs carrying mutations in *TP53*, which impart gain-of-function characteristics that promote metastasis. There is evidence that microRNAs (miRNAs) may play a role in both gain-of-function *TP53* mutations and metastasis, but this has not been fully explored in PDAC. Here we set out to identify miRNAs which are specifically dysregulated in metastatic PDAC. To achieve this, we utilised established mouse models of PDAC to profile miRNA expression in primary tumours expressing the metastasis-inducing mutant p53^R172H^ and compared these to two control models carrying mutations, which promote tumour progression but do not induce metastasis. We show that a subset of miRNAs are dysregulated in mouse PDAC tumour tissues expressing mutant p53^R172H^, primary cell lines derived from mice with the same mutations and in *TP53* null cells with ectopic expression of the orthologous human mutation, p53^R175H^. Specifically, *miR-142-3p* is downregulated in all of these experimental models. We found that DNA methyltransferase 1 (*Dnmt1*) is upregulated in tumour tissue and cell lines, which express p53^R172H^. Inhibition or depletion of *Dnmt1* restores *miR-142-3p* expression. Overexpression of *miR-142-3p* attenuates the invasive capacity of p53^R172H^-expressing tumour cells. *MiR-142-3p* dysregulation is known to be associated with cancer progression, metastasis and the miRNA is downregulated in patients with PDAC. Here we link *TP53* gain-of-function mutations to *Dnmt1* expression and in turn *miR-142-3p* expression. Additionally, we show a correlation between expression of these genes and patient survival, suggesting that they may have potential to be therapeutic targets.

## Introduction

PDAC is the most common and most aggressive form of pancreatic cancer and has very poor prognosis, with an average 5-year survival of <10%^1^. This is often due to the disease being diagnosed only after metastatic onset, and its resistance to conventional therapeutics^2^. A better understanding of the molecular events governing PDAC progression is required to develop more suitable therapeutic interventions.

Around 90% of sporadic human PDACs are initiated by mutations to the *K-RAS* proto-oncogene, which results in constitutive activation of K-RAS signalling^3^. Mouse studies with targeted mutation of *Kras* develop pancreatic intraepithelial neoplasia (PanIN), which progress slowly and lead to malignant adenocarcinomas at a low frequency^4^. The majority of mutant *Kras*-induced PanINs undergo senescence and require further mutations in tumour suppressors, such as *Trp53*^5,6^, *Cdkn2a*^7^, *Tgfbr2*^8^, *Smad4*^9^ or *Pten*^10,11^, to progress into invasive adenocarcinomas.

Mutations in *TP53* are present in ~40–70% of PDAC patients^12^. The majority of these mutations occur in the DNA-binding domain of *TP53*, resulting in a protein which lacks the capacity to bind DNA^13^. The mutated *TP53* proteins have dominant-negative effects on transcription as the insertion of just one mutated version into the *TP53* homo-tetramer prevents its function^14^. Additionally, a number of studies have shown that specific mutations in *TP53*, such as p53^R172H^, impart additional gain-of-function characteristics, including initiation of metastasis, which are not observed following *TP53* loss^5,6^.

Mutations to *PTEN* are not observed with high frequency in human PDACs, though hyper-activation of signalling pathways normally inhibited by *PTEN*, such as PI3K, is observed in ~60% of cases^15^. However, when they do occur, they allow cells to bypass *K-RAS*-induced senescence in PanIN lesions^11^ and thus promote formation of PDAC. Importantly, mutations to PTEN have not been shown to induce metastasis in PDAC^11^.

The incidence of the gain-of-function *TP53* mutations, including p53^R172H^ (R175H in humans), in aggressive tumours and their documented functional role in metastasis testifies to their importance, but their full molecular impact has not yet been elucidated^6,16–18^. A number of studies have strongly implicated miRNAs and their key biogenesis enzymes, Drosha and Dicer, as critical mediators of mutant *TP53* activity. Mutant *TP53* has been shown to interfere with Drosha interactions with DEAD-box protein 5 (DDX5/p68)^19^ and DEAD-box protein 17 (DDX17/p72/p82)^20^, leading to reduced processing of specific subsets of primary miRNAs. Moreover, previous studies have shown that decreased Dicer expression can result in metastasis^21,22^ and this process may involve members of the *TP53* family^23,24^. Mutant *TP53* has also been shown to interact with and inhibit other members of the *TP53* family: *TAp63* and *TAp73*^25^. In fact, miRNAs which are direct transcriptional targets of *TAp63*, *miR-130b*^26^ and *let-7i*^17^ have been shown to be downregulated by mutant *TP53*.

Together these studies strongly suggest that miRNAs are key mediators acting downstream of gain-of-function *TP53* mutations. MiRNAs relay their activity by negatively regulating gene expression through base pairing with hundreds of target mRNAs thus controlling gene expression networks and giving them the potential to affect complex cellular mechanisms such as metastasis^27^. Because of these observations, we wished to specifically focus on the miRNAs which were regulated in mouse models of PDAC carrying the gain-of-function mutant p53^R172H^. While miRNA expression profiles of PDAC in primary patient samples have previously been published^28^, none have looked specifically at how mutant *TP53* affects miRNA expression in this disease. This is critical given that mutant *TP53* has been shown to induce metastasis, which is the predominant cause of cancer-related morbidity and mortality. Here we show that mutant p53^R172H^ inhibits expression of *miR-142-3p*, a microRNA known to drive invasion and metastasis in PDAC^29^. We show that this occurs through a *DNMT1*-dependent mechanism leading to increased genomic methylation around the *miR-142-3p* genomic locus. Importantly, we show that both *DNMT1* and *miR-142-3p* expression correlate with patient survival, which may provide opportunities for therapeutic intervention.

## Results

### Mutant p53^R172H^ expression leads to downregulation of a subset of miRNAs in PDAC

The aim of this study was to identify miRNAs, which were dysregulated by the gain-of-function p53^R172H^ mutant in PDAC and thus potentially involved in metastasis as opposed to loss of p53 function, which does not cause metastasis^5,6^. *Pdx1-Cre; LSL-Kras*^*G12D/+*^*; LSL-Trp53*^*R172H/+*^ (KPC) mice carry pancreas-specific mutant Kras^G12D^ and gain-of-function p53^R172H^ and develop invasive and metastatic PDAC^5^. Tumour tissue from these mice (hereafter referred to as Kras p53^R172H^) was studied, along with tumour tissue from *Pdx1-Cre; LSL-Kras*^*G12D/+*^*; LSL-Trp53*^*flox/+*^ (Kras p53^flox^) mice and *Pdx1-Cre; LSL-Kras*^*G12D/+*^*; Pten*^*flox/+*^ (Kras Pten^flox^) mice, both of which develop PDACs that rarely metastasise^6,11^. The use of these two controls allowed us to differentiate between microRNAs whose expression is dependent on WT p53 and those regulated by the p53^R172H^ gain-of-function mutant. This is particularly important as previous studies have suggested that mutant p53^R172H^ gain-of-function is responsible for metastasis^5,6^. As Kras p53^flox^ cells have lower *Trp53* expression than Kras Pten^flox^ cells, microRNAs which show no change in expression between the controls but are significantly different in the Kras p53^R172H^ condition are likely to be changing due to gain of function rather than dominant-negative activity of mutant p53^R172H^. Global miRNA expression profiles were investigated using miRNA microarrays, interrogating total RNA from mouse primary PDAC tissues.

**Fig. 1:**
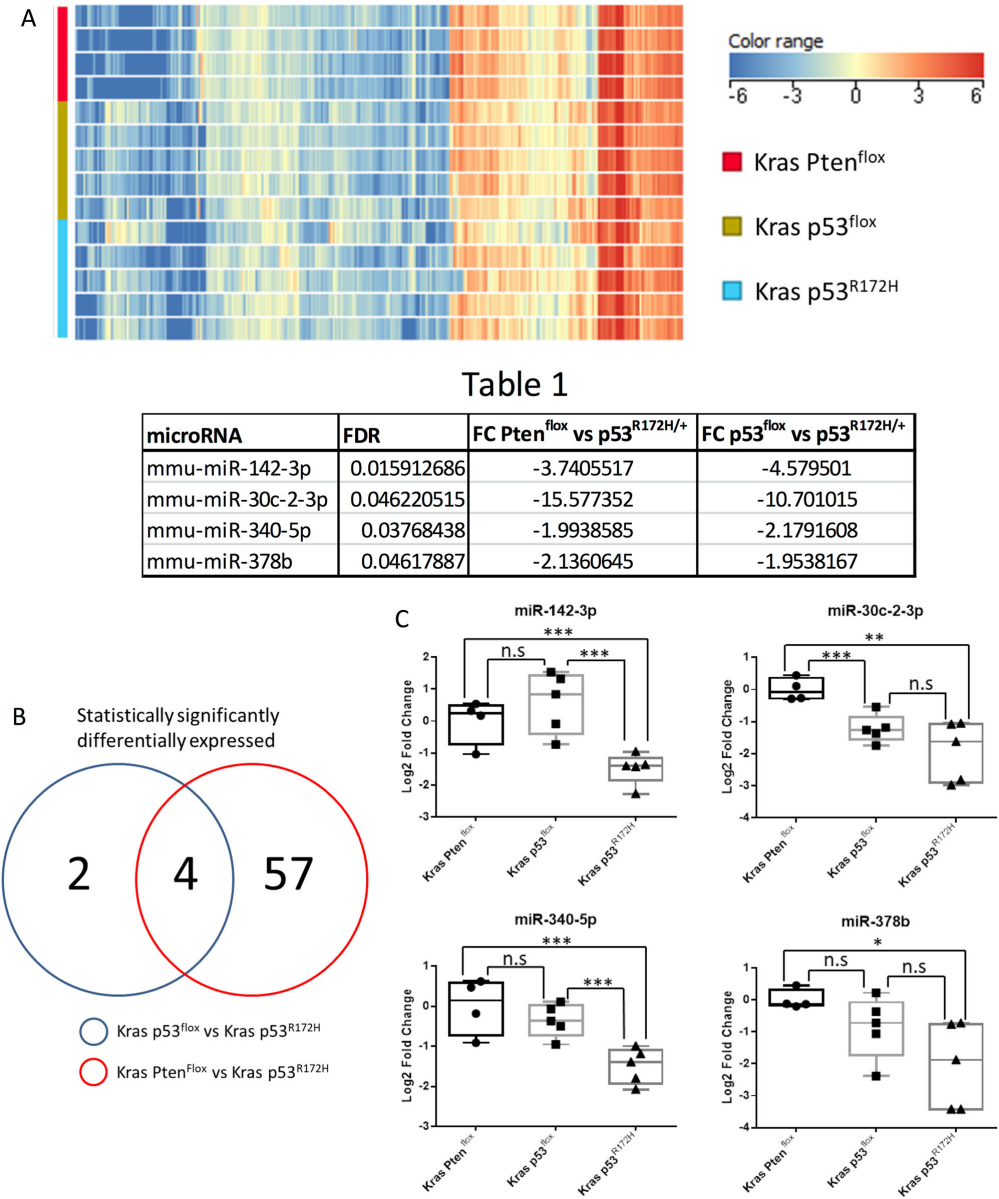
Global profiling of microRNA expression in tissues from PDAC mouse models. **a** Agilent microRNA microarrays were used to investigate global microRNA expression profiles from Kras Pten^flox^ (*n* = 4), Kras p53^flox^ (*n* = 5) and Kras p53^R172H^ (*n* = 5)-expressing mouse pancreatic tumour tissues. Heat map shows the hierarchal clustering and expression levels of microRNAs for all the samples in the analysis. Statistical analysis and fold changes presented in Table 1 were calculated using a one-way ANOVA with post hoc analysis using Tukey’s honest significance difference test (HSD) with an FDR (Benjamini Hochberg)-adjusted *p* value cutoff of 0.05. A list of all statistically significantly changed microRNAs can be found in supplemental Fig. 1. **b** Venn diagram demonstrating the relationship between miRNAs differentially expressed in Kras p53^R172H/+^ vs Kras Pten^flox^ and Kras p53^R172H/+^ vs Kras p53^flox^ tumours. **c** RT-qPCR was used to validate the microarray data using the ΔΔCT method with U6 snRNA as the reference gene. A two-sample, two-tailed, unpaired *t*-test was used to compare the ΔCT values from each group. Statistical significance is represented as *p < 0.05, **p < 0.01 and ***p < 0.005

MiRNAs were included in the study if all of the samples within at least one of the conditions had detectable signals for it in the microarray. Using this cutoff, a total of 307 miRNAs were detected (Fig. 1a). Statistical analysis identified 61 miRNAs which were significantly differentially expressed in the Kras p53^R172H^ samples compared to the Kras Pten^flox^ samples, and six miRNAs which were significantly differentially expressed in the Kras p53^R172H^ samples compared to the Kras p53^flox^ samples (Supplemental Fig. 1). A total of four miRNAs, *miR-142-3p*, *miR-30c-2-3p*, *miR-340-5p* and *miR-378b*, were found to be dysregulated in the Kras p53^R172H^ tissues compared to both the Kras p53^flox^ and Kras Pten^flox^ tissues. All four were downregulated in the Kras p53^R172H^ tissues (Fig. 1b and Table 1). RT-qPCR validation confirmed that the expression of *miR-142-3p* and *miR-340-5p* does not differ between the two control conditions (Kras p53^flox^ and Kras Pten^flox^), but both miRNAs are significantly downregulated in the Kras p53^R172H^ tissues (Fig. 1c). Expression of miR-30c-2-3p and miR-378b was found to be significant between Kras Pten^flox^ and Kras p53^R172H^ but not between Kras Pten^flox^ and Kras p53^R172H^ (Fig. 1c). This suggests that *miR-142-3p* and *miR-340-5p* expression is directly affected by mutant p53^R172H^ expression.

**Table 1:** ▓▓▓▓

**microRNA**	**FDR**	**FC Pten** ^**flox**^ **vs p53** ^**R172H**^	**FC p53** ^**flox**^ **vs p53** ^**R172H**^
mmu-miR-142-3p	0.015912686	−3.7405517	−4.579501
mmu-miR-30c-2-3p	0.046220515	−15.577352	−10.701015
mmu-miR-340-5p	0.03768438	−1.9938585	−2.1791608
mmu-miR-378b	0.04617887	−2.1360645	−1.9538167

**Fig. 2:**
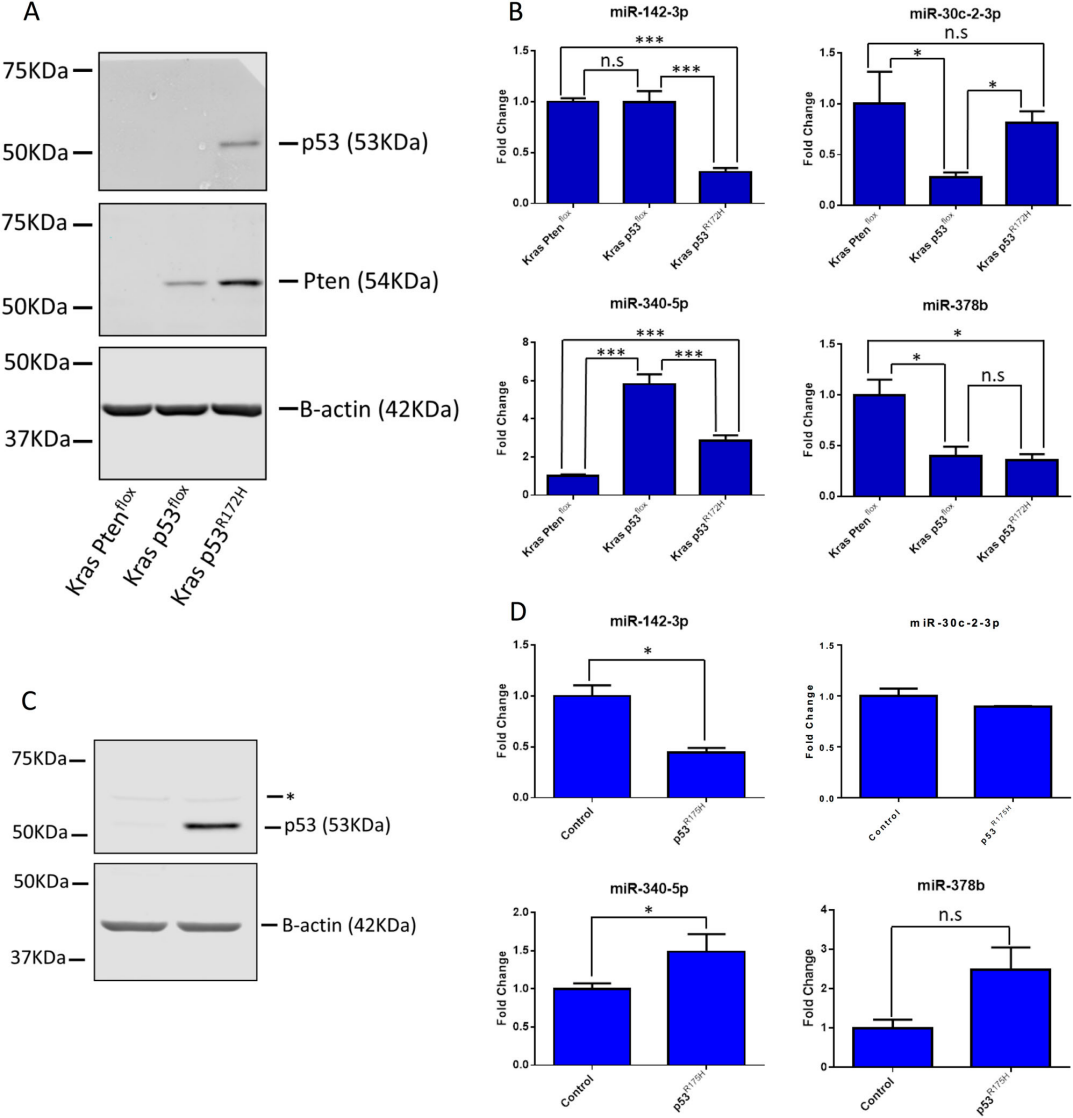
Validation of microRNA expression in primary cell lines and Kras p53^flox^ cells with ectopic expression of mutant p53^R172H^. **a** Primary cell lines with the same genotype as the primary tissue samples used in Fig. 1 were analysed for protein expression by western blot. **b** The expression of the microRNAs identified as being dysregulated in p53^R172H^-expressing tissues was investigated in these cell lines using the ΔΔCT method with U6 snRNA as the reference gene. The results represent the mean of three biological repeats; the error bars represent the maximum relative quantity. A two-sample, two-tailed, paired *t*-test was used to compare the ΔCT values from each group. Statistical significance is represented as *p < 0.05 and ***p < 0.005. **c** Kras p53^flox^ with ectopic expression of p53^R175H^ and a control cell line stably expressing an empty vector were analysed by western blot. The * represents a non-specific band of unknown origin. **d** Expression of the microRNAs identified as being dysregulated in the Kras p53^R172H^ tissues was interrogated by RT-qPCR in these cell lines using the ΔΔCT method with U6 snRNA as the reference gene. The results represent the mean of three biological repeats with the error bars representing the maximum relative quantity. A two-sample, two-tailed, paired *t*-test was used to compare the ΔCT values from each group. Statistical significance is represented as *p < 0.05

This analysis provides information about miRNA expression changes in the physiological context of the primary tumour environment in mouse models. However, tissues are inherently heterogeneous with respect to cellular populations. In order to test whether the miRNAs dysregulated in the Kras p53^R172H^ tissues were specifically downregulated in tumour cells, we employed primary cell lines derived from each of the tumour models. As expected, Kras Pten^flox^ cell lines do not express PTEN, consistent with LOH during tumour progression as described previously^11^, and similarly Kras p53^flox^ cell lines do not express p53 as the remaining wild-type allele is lost during tumourigenesis (Fig. 2a). Thus, p53 was only observed in Kras p53^R172H^ lines, where the mutant protein is insensitive to degradation (Fig. 2a). RT-qPCR of the cells lines showed that expression of *miR-142-3p* follows the profile observed in the tissue samples, with no difference between the control conditions (Kras p53^flox^ and Kras p53^R172H^) and a significant downregulation in the mutant Kras p53^R172H^ cells (Fig. 2b). The expression of the other miRNAs identified in tissue samples did not show the same pattern of changes which might indicate that the observed differences in tissue may be due to their heterogeneity (Fig. 2b).

Next, we used a murine Kras p53^flox^ cell line with ectopic expression of the analogous human mutant TP53^R175H^ to show that the human orthologue of this mutation was also capable of affecting the expression of the miRNAs of interest^6^. As tumours in Kras p53^flox^ mice undergo loss of the remaining wild-type *Trp53* allele, wild-type p53 is not expressed in these cells. This allowed us to exclude that the observed changes in miRNA expression are due to the dominant-negative effect of mutant p53 on wild type. Western blot analysis confirmed that *TP53* is only present in the cell line with ectopic expression of mutant p53^R175H^ (Fig. 2c). Ectopic expression of mutant p53^R175H^ led to a downregulation of *miR-142-3p*, which confirmed our results for the mouse protein. No change was observed for *miR-30c-2-3p* and expression of *miR-340-5p* and *miR-378b* were increased (Fig. 2d), again indicating that the altered expression of these miRNAs is not a reproducible hallmark of mutant p53 expression in isolated tumour-derived cells. As there is no wild-type p53 expressed in these cells for the mutant p53^R175H^ to act upon in a dominant-negative manner, this strongly suggests that *miR-142-3p* is being downregulated due to the gain-of-function activity of this mutant. In addition, these data show that the human orthologue of the *TP53* mutant is also able to affect the expression of *miR-142-3p*.

### Dnmt1 is dysregulated by mutant p53^R172H^ in PDAC

**Fig. 3:**
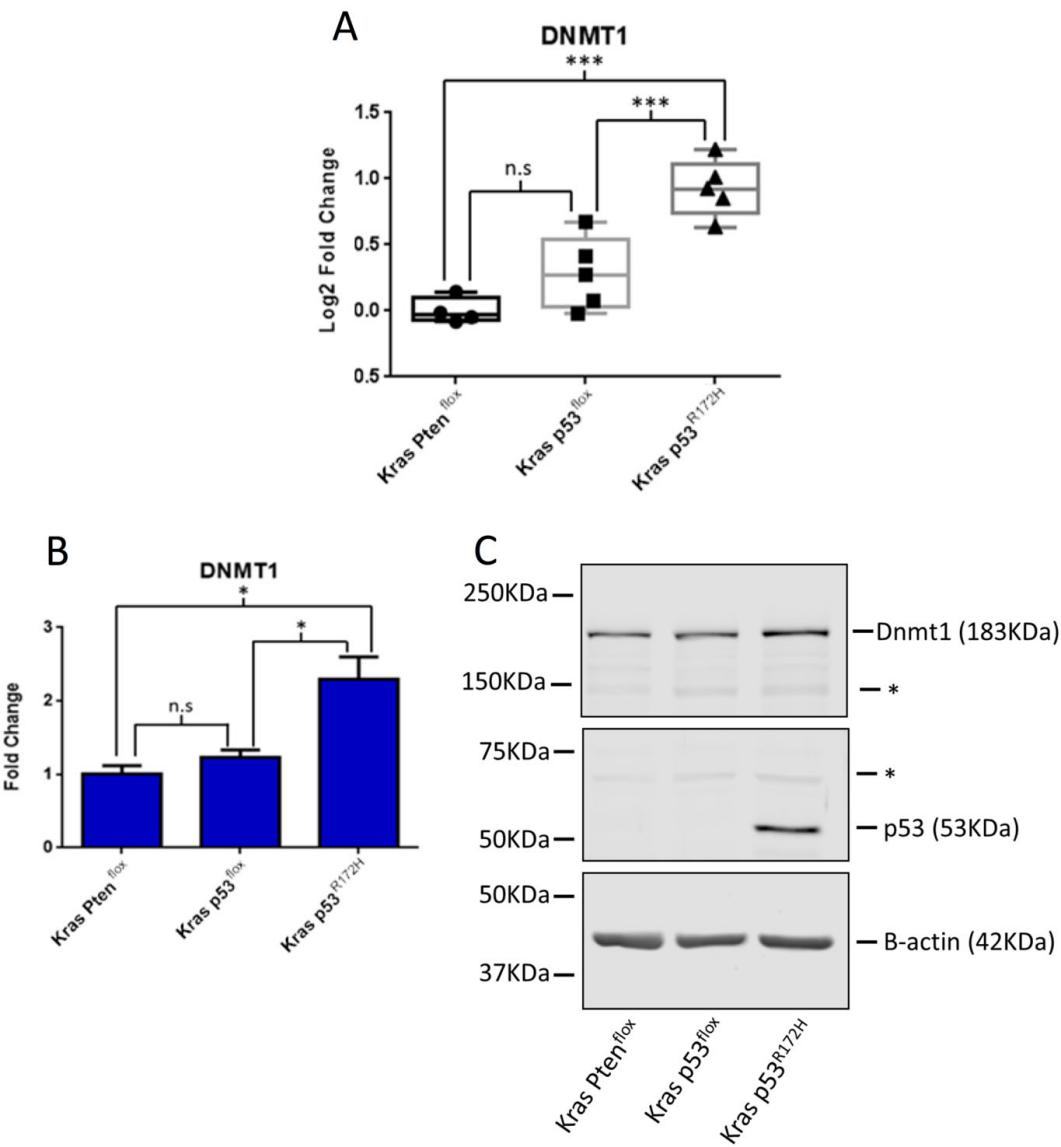
Protein and mRNA expression of Dnmt1 in PDAC mouse models. **a** RT-qPCR of *Dnmt1* mRNA abundance in primary mouse tissues. The ΔΔCT method was used to calculate fold change using β-actin as the reference gene with the fold change being relative to the Kras Pten^flox^ samples. The * represent non-specific bands of unknown origin. **b** RT-qPCR of *Dnmt1* expression in primary cell lines. The ΔΔCT method was used to calculate fold change with β-actin as the reference gene with fold change normalised to the Kras Pten^flox^ cell line. The result represents the mean of three biological repeats with the error bars representing the maximum relative quantity. A two-sample, two-tailed, paired *t*-test was used to compare the ΔCT values from each group. Statistical significance is represented as *p < 0.05 and ***p < 0.005. **c** A representative western blot showing the expression of *Dnmt1*, *Pten*, *p53* and β*-actin* in the primary cell lines

Our investigations of the PDAC models did not uncover any global changes in mature miRNA expression, so we focused on regulatory mechanisms pertaining to the specific dysregulation of *miR-142-3p* expression. Since DNA methylation has been found to be both upregulated^30–33^ and downregulated^34–36^; in a number of cancer types including PDAC, we reasoned that dysregulated methylation may be responsible for the observed changes in *miR-142-3p* expression. *Dnmt1* mRNA expression was investigated in the primary tumour tissue from the PDAC models and was found to be elevated in tumours from the Kras p53^R172H^ tissues compared to both Kras p53^flox^ and Kras Pten^flox^ tissues (Fig. 3a). *Dmnt1* mRNA (Fig. 3b) and protein (Fig. 3c) were also found to be upregulated in the Kras p53^R172H^ cell line compared to both the Kras p53^flox^ and Kras Pten^flox^ cell lines.

### MiR-142-3p expression can be rescued by inhibition of methylation

**Fig. 4:**
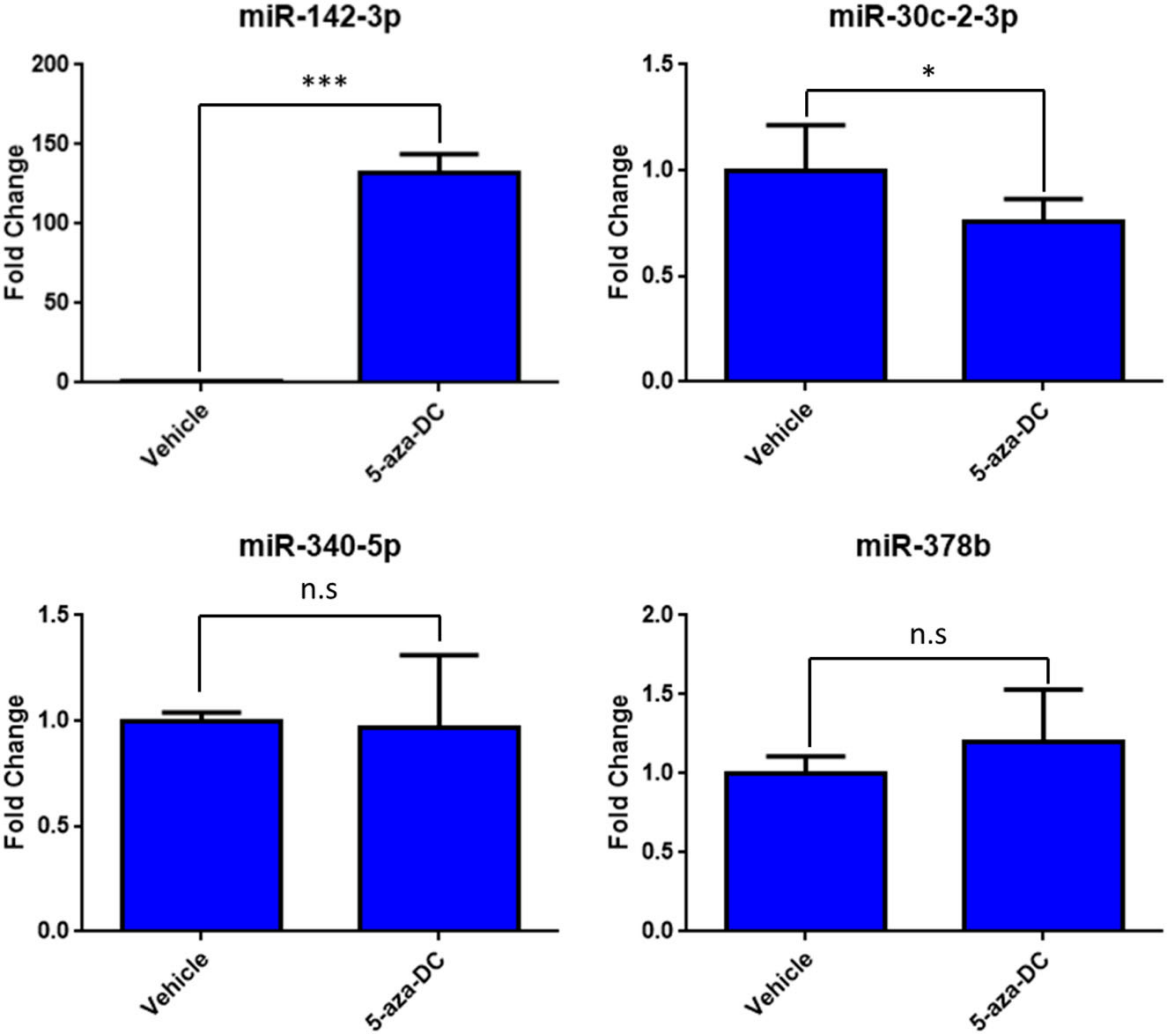
Expression of microRNAs following treatment with 5-aza-deoxycytidine. The Kras p53^R172H/+^ cell line was treated with 1 µm 5-aza-deoxycytidine (5-aza-Dc) or an equal volume of a vehicle control (DMSO) for 24 h. The expression of the microRNAs identified as dysregulated in p53^R172H^-expressing tissues was quantified by RT-qPCR with U6 snRNA as the reference gene. The result represents the average of three biological repeats with the error bars representing the maximum relative quantity. A two-sample, two-tailed, paired *t*-test was used to compare the ΔCT values from each group. Statistical significance is represented as *p < 0.05 and ***p < 0.005

**Fig. 5:**
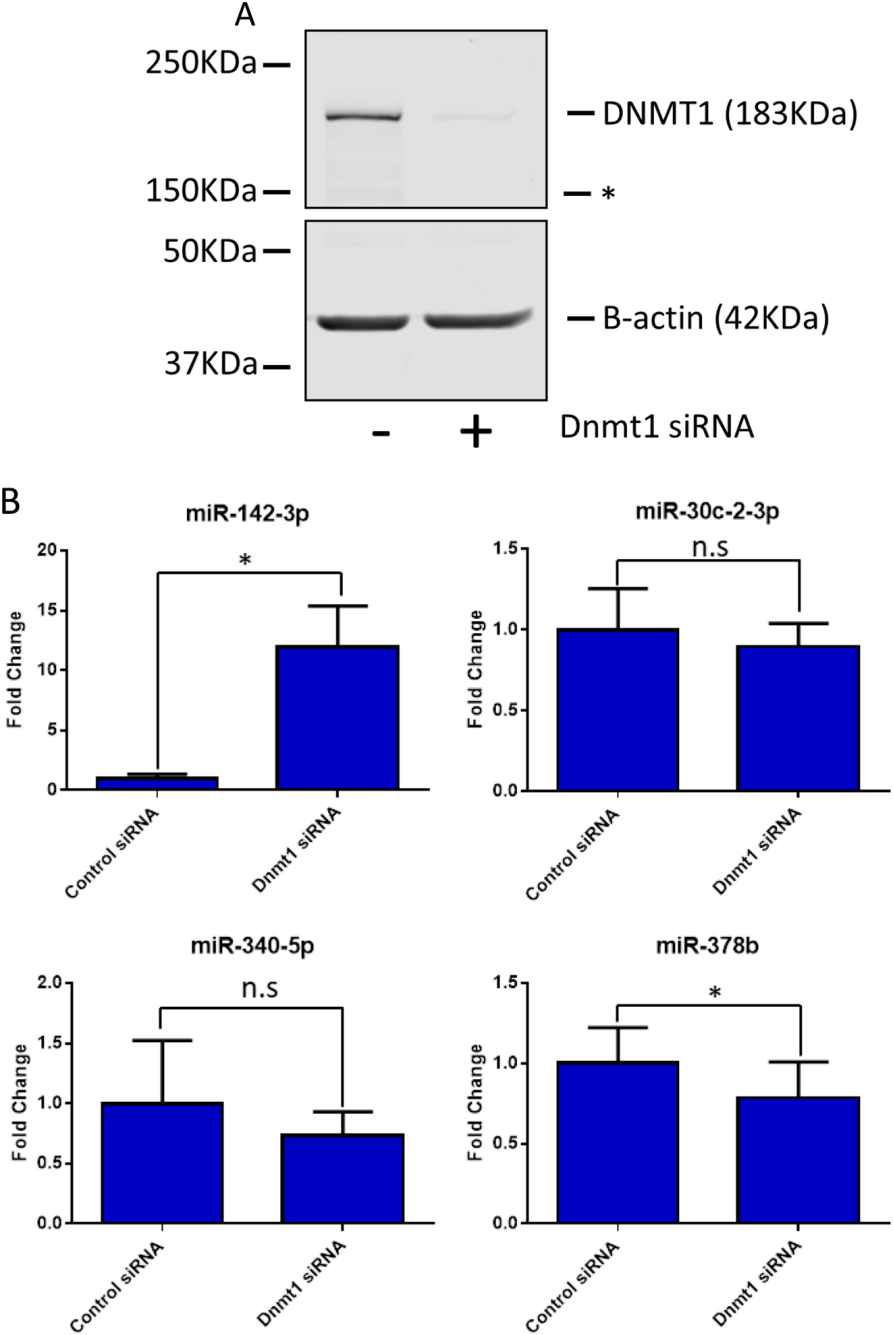
Expression of microRNAs following depletion of *Dnmt1*. The Kras p53^R172H^ cell line was treated with 20 nM DNMT1 siRNA or a control siRNA for 48 h. **a** A representative western blot showing the expression of *Dnmt1* and *p53* following *Dnmt1* depletion with β-actin as a loading control. The * represents a non-specific band of unknown origin. **b** The expression of the microRNAs dysregulated in p53^R172H^-expressing tissues was quantified after *Dnmt1* depletion by RT-qPCR with U6 snRNA as the reference gene. The result represents the mean of three biological repeats with error bars representing the maximum relative quantity. A two-sample, two-tailed, paired *t*-test was used to compare the ΔCT values from each group. Statistical significance is represented as *p < 0.05

As we observed an inverse correlation between *miR-142-3p* and *Dnmt1* expression, we asked if inhibiting DNA methylation had an impact on *miR-142-3p* expression levels. We utilised 5-aza-2-deoxycytidine (5-aza-DC), which inhibits DNMT1, DNMT3A and DNMT3B by being incorporated into nascent DNA, where it cross links with *DNMT* protein family members, sequestering them and reducing global methylation^37^. Treatment of the Kras p53^R172H^ cell line for 24 h with 1 μM of 5-aza-DC led to a significant induction of *miR-142-3p* while having no effect on the expression of other miRNAs investigated in this study (Fig. 4). As 5-aza-DC is a functional inhibitor of all members of the DNMT family^38^, we asked if specific inhibition of *Dnmt1* was also able to affect *miR-142-3p* expression. As in the case of 5-aza-DC treatment, expression of *miR-142-3p* was significantly increased following depletion of *Dnmt1* in the Kras p53^R172H^ cell line (Fig. 5a, b). The other miRNAs under investigation showed mild to no change in expression following *Dnmt1* depletion, demonstrating selectivity for *miR-142-3p*.

### The *miR-142* genomic locus is hypermethylated in mutant p53^R172H^-expressing primary cell lines

**Fig. 6:**
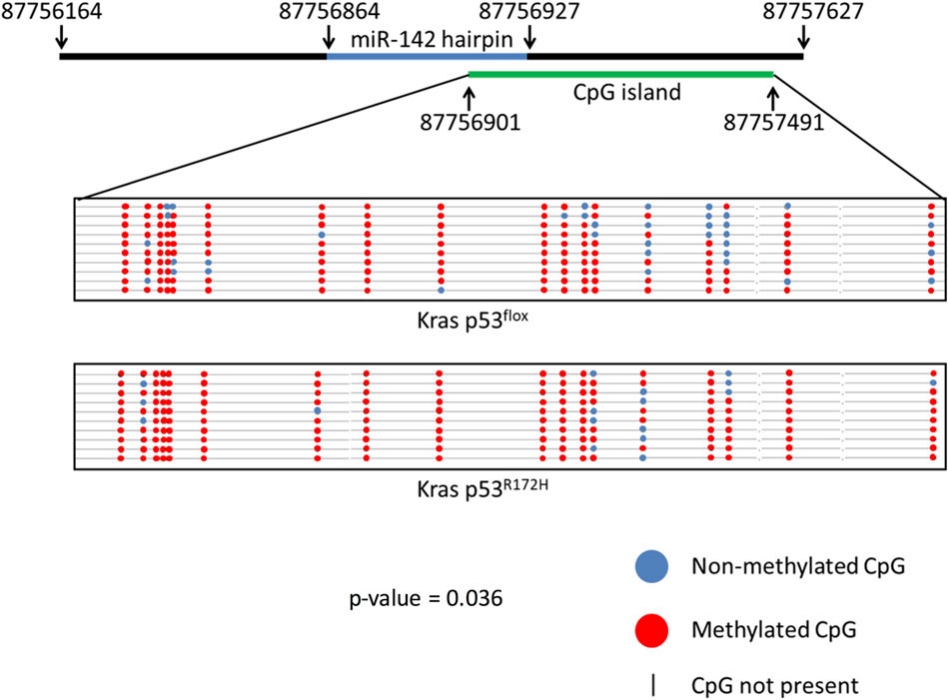
Bisulphite sequencing reveals increased methylation around the miR*-142* genomic locus in the Kras p53^R172H^ cell line. **a** 378 bp fragment of a CpG island which contains 18 CpGs and overlaps the *miR-142* genomic locus was amplified and investigated using bisulphite sequencing. A schematic shows the location of the CpG island. The lollipop plot shows the location of the CpGs with black spots representing methylated CpGs and white spots representing non-methylated CpGs (**a**). A two-tailed Mann–Whitney *U-*test was used to assess for statistically significant differences between the two bisulphite sequencing groups

As direct depletion of *Dnmt1* and a methylation-inhibiting drug were both shown to induce *miR-142-3p* expression, direct analysis of CpG dinucleotides in the *miR-142* genomic locus was carried out. A CpG island which overlaps the *miR-142* genomic locus was identified using Methyl Primer Express software (Applied Biosystems, Waltham, MA, USA) (Fig. 6). Bisulphite sequencing of a 378 bp fragment of the CpG island was carried out using DNA from both the Kras p53^flox^ and Kras p53^R172H^ cell lines and clearly showed high levels of methylation of this region in both cell lines. Importantly, a statistically significant increase in methylation was observed in the fragment from the Kras p53^R172H^ cell line (Fig. 6).

### Overexpression of miR-142-3p inhibits p53^R172H^-driven invasion in vitro

**Fig. 7:**
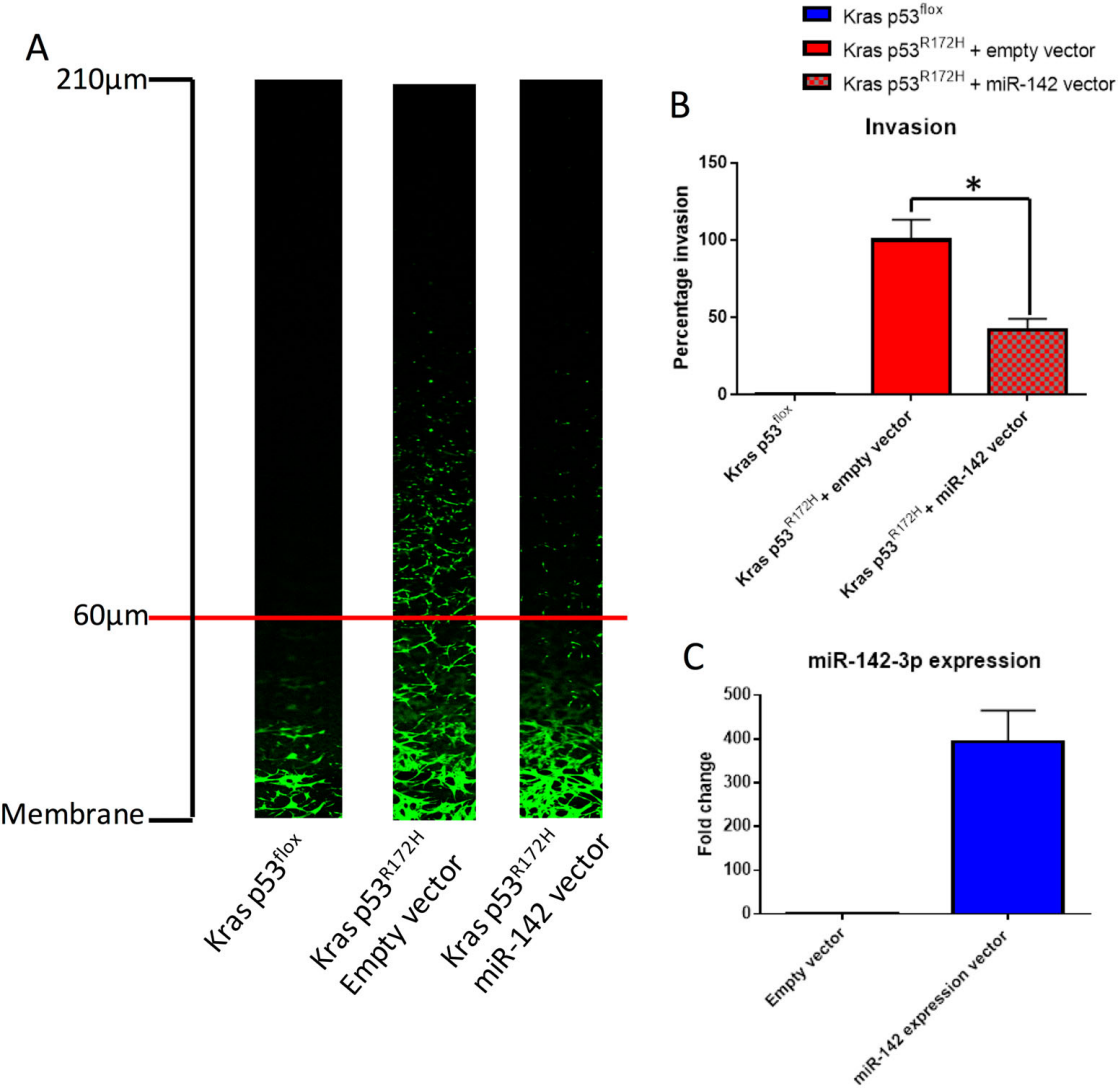
miR-142-3p overexpression inhibits mutant p53-driven invasion in vitro. **a** Representative Z-stack images from reverse transwell invasion assays. Kras p53^flox^ (*n* = 2), Kras p53^R172H^ + empty vector (*n* = 4) and Kras p53^R172H^ + miR-142 expression vector (*n* = 4) mouse primary PDAC cells were analysed for their invasive potential. Cells were considered invasive if they migrated further than 60 μm. **b** Quantification of invasion by measuring the fluorescence of cells past 60 μm relative to the total fluorescence of each stack. Each experimental repeat consisted of two transwells each with three Z-stacks per transwell. The percentage invasion is reported relative to the Kras p53^R172H^ cells transfected with an empty vector. Error bars represent the pooled standard deviation for each condition. A two-sample, two-tailed, paired *t*-test was used to compare the average percentage of invasion between biological repeats (*n* = 4) for Kras p53^R172H^ + empty vector and Kras p53^R172H^ + miR-142-3p expression vector. Statistical significance is represented as *p < 0.05. **c** The average change in miR-142-3p expression in cells transfected with the miR-142-3p expression vector compared to cells transfected with empty vector. Error bars represent the standard deviation

Given that mutant p53^R172H^ has been shown to drive invasion and metastasis in PDAC^6^, we investigated the impact of overexpression of miR-142-3p on cell invasion. Using reverse transwell invasion assays^6^, we confirmed that Kras p53^flox^ cells have almost no invasive ability while Kras p53^R172H^ cells are invasive (Fig. 7a, b). This is in line with previously published data^6^. Importantly, overexpression of miR-142-3p in the Kras p53^R172H^ cells leads to a significant reduction in the invasive potential of these cells in vitro (Fig. 7a–c).

### Low *miR-142-3p* expression and high DNMT1 expression correlate with poor survival in human cancers

The data we present here identify a novel mechanism by which mutant p53^R172H^ is able to decrease expression of *miR-142-3p* in a mouse model of PDAC, through genomic hypermethylation due to increased expression of *DNMT1*. Therefore, we wished to determine whether *miR-142-3p* or *DNMT1* expression correlates with human patient survival.

**Fig. 8:**
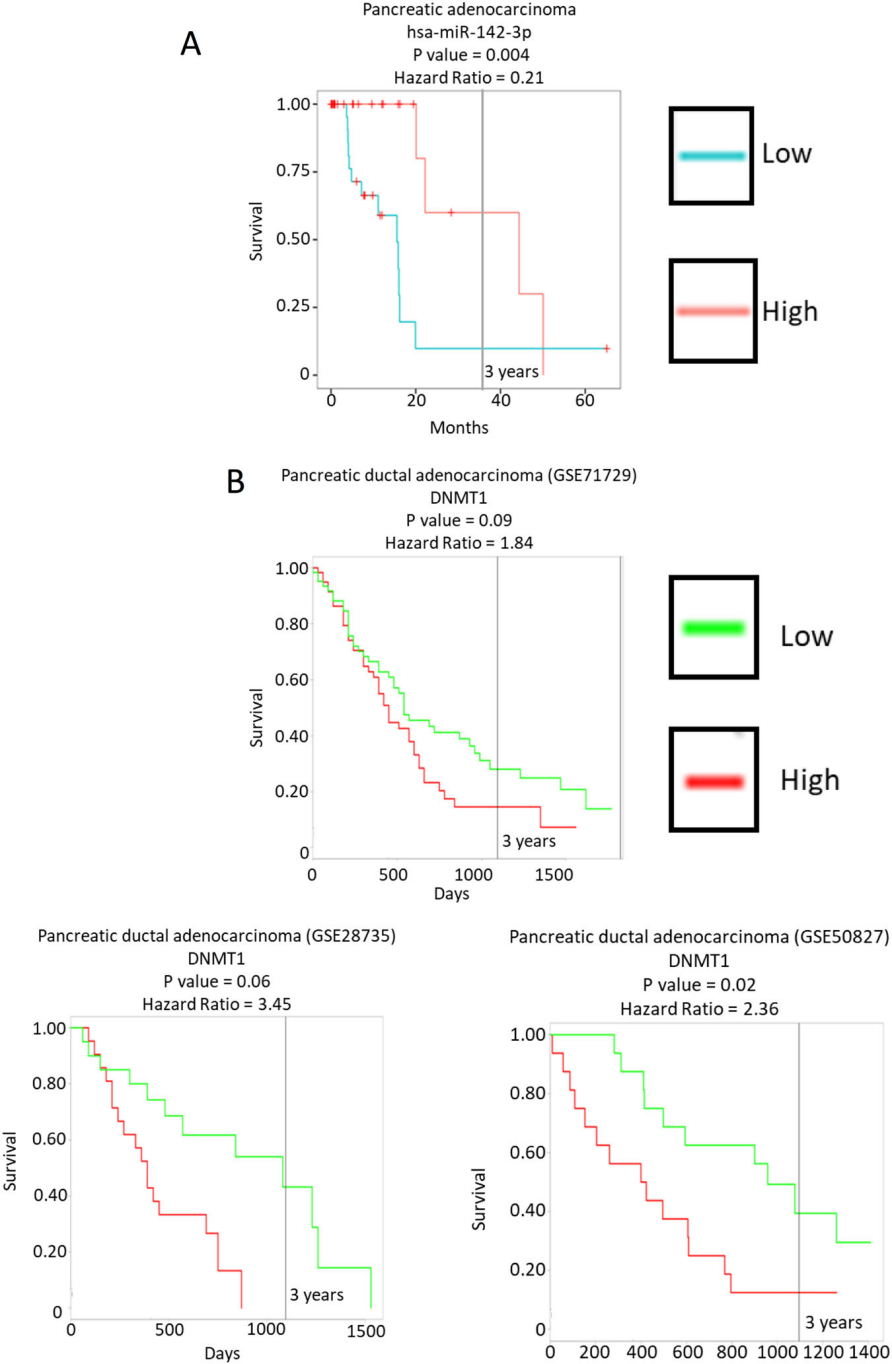
Low *miR-142-3p* and high *DNMT1* correlate with a poor prognosis in human PDAC. **a** The data set in the YM500v3 database shows a clear correlation between low *miR-142-3p* expression and poor survival in human PDAC. **b** The ProGENEV2 database was interrogated for correlations between *DNMT1* expression and human PDAC patient survival. All data sets which show a statistically significant correlation are reported, all of which show a correlation between high *DNMT1* expression and poor patient survival

Bioinformatic analysis using the YM500v3 database allowed us to examine 8000 small RNA sequencing data sets and correlate survival statistics associated with their expression^39^. This revealed a strong correlation between poor survival and low *miR-142-3p* expression in human PDAC (Fig. 8a). The ProGENEV2 database^40^ was used to investigate any correlation between *DNMT1* expression and patient survival in PDAC. This revealed that high *DNMT1* mRNA expression correlates with poor survival in human PDAC patients (Fig. 8b).

## Discussion

Three mouse models of PDAC were used in this study: two in which metastasis occurs only at low-frequency Kras p53^flox^ and Kras Pten^flox^, and Kras p53^R172H^, in which metastasis occurs at high frequency. Murine primary pancreatic tumour tissues and primary cell lines, as well as ectopic expression of human mutant p53^R175H^ in *TP53* null cells, all showed that *miR-142-3p* is downregulated in mutant p53^R172H^ or p53^R175H^-expressing PDAC.

A recent study found that *miR-142-3p* is downregulated in human PDAC when compared to paired adjacent normal tissue and in pancreatic cancer cell lines, and showed that hypoxia-inducible factor (*HIF1α*) is a direct target of *miR-142-3p* in PDAC^29^. High *HIF1α* expression leads to increased proliferation and invasion of tumour cells as well as inducing expression of genes involved in EMT, such as *vimentin*, *VEGF* and *E-cadherin*. Meanwhile, low expression of *miR-142-3p* correlated with an increased incidence in lymphatic metastasis. These results taken together with our findings identify both direct and downstream secondary targets by which *miR-142-3p* functions in human patients and link its expression to invasion and metastasis. Additionally, two recent studies have shown that increasing *miR-142-3p* expression in acute myelogenous leukaemia^41^ and non-small cell lung cancer^42^ improves chemosensitivity by decreasing autophagy due to translational repression of *HMGB1*. However, the previously published data does not connect dysregulation of *miR-142-3p* to gain-of-function mutations in *TP53* nor does it identify a mechanism by which *miR-142-3p* is dysregulated. Our data clearly show that the expression of this miRNA is indirectly inhibited by mutant p53^R172H^ via genomic methylation due to increased expression of *DNMT1*.

Our study also found that mutant p53^R172H^ elevates the expression of *Dnmt1* in PDAC. This is in line with previous data, which has found DNMT1 to be upregulated in both PanIN lesions and PDACs^43,44^. Our data show that *Dnmt1* mRNA is upregulated in mutant p53^R172H^-expressing tissues and cell lines, suggesting an increase in transcription rate or stabilisation of the *Dnmt1* transcript. *DNMT1* has been shown to be a transcriptional target of *specificity protein 1 & 3* (*SP1 & 3*)^45^. A complex formed between *SP1* and wild-type *TP53* has been shown to bind the promoter of *DNMT1* and repress its transcription^46^. Mutant p53 is able to inhibit this complex resulting in upregulation of *DNMT1* in lung cancer patients^46^. It is possible that the same mechanism is inducing *Dnmt1* transcription in this PDAC model, leading to subsequent hypermethylation of *miR-142-3p*.

Previous findings of the impact of *miR-142-3p* on tumour progression along with the data presented in this study, linking mutant p53^R172H^ and *DNMT1* expression, suggest that *DNMT1* may be a potential therapeutic target in PDAC. However, demethylating drugs have been shown to have limited clinical efficacy. 5-aza-deoxycytidine has previously been considered in cancer treatment but was shown to be highly toxic^47^, though more recent studies using low dosage of 5-aza-deoxycytidine have demonstrated some clinical efficacy in haematological malignancies and myelodysplastic syndrome^48–52^. A previous study showed that treatment with 5-aza-deoxycytidine in PDAC leads to upregulation of tumour suppressors, but also increased expression of oncogenes which promote metastasis^53^, limiting its use as a therapeutic agent. Interestingly, specifically ablating *DNMT1* promotes expression of tumour suppressor genes but does not have the undesirable effect of promoting expression of oncogenes^54^. A number of drugs which directly target *DNMT1* are currently in development, though most show limited therapeutic value^55^. One interesting drug is minnelide, a pro-drug of triptolide. Triptolide has been shown to be efficacious in treatment of PDAC through induction of *miR-142-3p*^56^. Another study has shown that triptolide is able to negatively regulate *DNMT1* transcription, leading to increased expression of methylated genes^57^. These findings are very much in line with our data which show that *Dnmt1* inhibition increases *miR-142-3p* expression and that increasing *miR-142-3p* expression reduces the invasive potential of tumour cells. This may suggest that triptolide may be specifically useful in treatment of mutant *TP53*-expressing PDACs.

The data presented in this study identifies a potential new mechanism by which mutant p53^R172H^ is able to affect gene expression in a gain-of-function manner, through increased expression of *DNMT1* which in turn leads to hypermethylation of *miR-142-3p* and perhaps other genes. These results open up the possibilities for therapeutic targeting of methylation in mutant p53^R172H^ expressing PDACs and other tumour types.

## Methods

### Mouse models

All mouse models have been previously described^5,6,11,58^. Experiments were performed under Home Office licence and approved by the University of Glasgow ethics committee. Mice on a mixed background were maintained in conventional cages with environmental enrichment on a light-dark cycle and given access to standard diet and water ad libitum. Genotyping was performed by Transnetyx (Cordoba, TN, USA). Mice were monitored at least three times weekly and culled when exhibiting symptoms of PDAC (swollen abdomen, loss of body conditioning reminiscent of cachexia, jaundice, hunching and immobility). PDAC tissue was collected and stored at −80 °C in RNAlater (Ambion, Foster City, CA, USA) until RNA extraction. PDAC cell lines from these models were isolated and grown in house as described previously^6,58^.

### RNA extraction

Tissues were thawed on ice in the presence of RNAlater. Once thawed, tissues were snap frozen in liquid N_2_ and pulverised in a pestle and mortar before 1 ml of TRIzol (Thermo Fisher Scientific, Waltham, MA, USA) reagent per 1 mg of tissue was added. Chloroform (200 μl per 1 ml of TRIzol) was then added and the sample was centrifuged at 12,000 × *g* for 15 min and the aqueous layer was removed. Isopropanol (750 μl per 1 ml of TRIzol) was then added and the sample was precipitated overnight at −20 °C. RNA was resuspended in ddH_2_O and an equal volume of acid phenol/chloroform (50:50) was added. The sample was centrifuged at 12,000 × *g* and the aqueous layer was removed before sodium acetate (10% of final volume) and ethanol (250% of final volume) was added and the sample was precipitated at −20 overnight. Samples were resuspended in a suitable volume of ddH_2_O and were quantified using a nanodrop spectrophotometer (Thermo Fisher Scientific).

### MiRNA microarrays

Agilent miRNA microarrays were carried out as per the manufacturer’s instruction using the Agilent miRNA microarray labelling kit (Agilent, Santa Clara, CA, USA). In brief, 100 ng of total RNA from each PDAC tumour tissues was dephosphorylated and labelled with Cy3-pCp using T4 RNA ligase. The labelled RNA for each sample was then applied to one of the eight miRNA microarrays on each slide and sealed with a gasket cover, before being placed in a hybridisation oven at 55 °C for 20 h while rotating at 20 rpm. The labelled microarrays were scanned using the Agilent G2565CA microarray scanner and the features were defined and extracted using Agilent feature extraction software (version 11.0) using default settings. All fold change and statistical analysis was carried out using Genespring (Agilent). All non-mouse miRNAs were removed from the analysis along with any miRNA, which was not detected in all samples from at least one condition. Statistical analysis was carried out using a one-way ANOVA with a false discover rate (FDR) *p* value of 0.05 and post hoc analysis using Tukey’s honest significant difference test (HSD).

### Taqman RT-qPCR

RT-qPCR of miRNAs was carried out using Taqman miRNA assays (Applied Biosystems) as per the manufacturer’s instruction. In brief, 100 ng of total RNA was reverse transcribed using the TaqMan miRNA reverse transcription kit (Applied Biosystems). RT-qPCR was achieved using the Applied Biosystems 7500 RT-qPCR thermocycler under default conditions. The following Taqman assays were used in this study: *U6* – 001973, *miR-142-3p* – 000464, *miR-30c-2-3p* – 002110, *miR-340-5p* – 002258 and *miR-378b* – 465314_mat.

### SYBR green RT-qPCR

Reverse transcription for RT-qPCR of mRNAs was achieved using Superscript 3 (Invitrogen) reverse transcriptase and random primers as per the manufacturer’s instruction. Aliquot of 100 ng of total RNA was used for each reaction. The following primers were used in this study: *Dnmt1* forward – GTGCTCTCACCCAGAGCCCC,*Dnmt1* reverse – GGGTGCTTGACAGAAGCGCT, *β-actin* forward – GTGGACAGTGAGGCCAGGAT, *β-actin* reverse – GATTACTGCTCTGGCTCCTAGCA.

### Bisulphite sequencing

DNA was extracted from Kras p53^flox^ and Kras p53^R172H^ cell lines using the DNeasy blood and tissue kit (Qiagen, Venlo, the Netherlands) as per the manufacturer’s instruction. DNA was treated with sodium bisulphite using the EpiTect plus DNA bisulphite kit (Qiagen) as per the manufacture’s instruction. Following bisulphite conversion, DNA was washed and eluted from a MinElute DNA spin column. Methyl primer Express software (Applied Biosystems) was used to interrogate a sequence of DNA 700 bp both up and downstream of the miR-142 precursor sequence. Using the definition of a CpG island as a sequence with a CpG observed/expected ratio >0.6, a 590 bp CpG island was observed overlapping the miR-142-3p precursor sequence. Primers were designed to amplify a 378 bp fragment of the CpG island which contained 18 CpG dinucleotides.

PCR was carried out using EpiMark Hot Start Taq DNA Polymerase (NEB) using the following primers: forward – TGGATGAGTGTATTGTGGGT and reverse – AACCCCAATAACAAAATCAAAC as per the manufacturer’s instruction. The resulting fragment was size excluded on an agarose gel and extracted, before being ligated into pGEM-T Easy (Promega, Madison, WI, USA). The resulting ligation was transformed into DH5α competent cells and selected using blue white screening. A total of ten white colonies were selected and amplified for each Kras p53^flox^ and Kras p53^R172H^ cell line. DNA from the amplified colonies was prepared using Wizard Plus SV Miniprep kit (Promega) and Sanger sequenced using T7 and SP6 primers. A consensus sequence was then created using SeqTrace^59^. Consensus sequences were edited to remove and sequence corresponding to the pGEM-T Easy backbone using BioEdit^60^. The sequences were then compared to the genomic sequence of the CpG island using BiQ Analyzer^61^. Samples were accepted as individual if they had at least one non-CpG cytosine residue independent of the other samples. Samples were only accepted if they had a conversion efficiency of >95% judged by abundance of non-CpG cytosine residues.

### SDS-PAGE and western blotting

Cells were lysed in radioimmunoprecipitation assay buffer (RIPA) (50 mM Tris, 150 mM NaCl, 0.1% SDS, 0.5% sodium deoxycholate, 1% NP-40) supplemented with Complete Protease Inhibitor Cocktail Tablets (Roche, Basel, Switzerland) immediately prior to use. The antibodies used in this study were: β-actin (Sigma, 20-33), PTEN (Cell Signalling, 9552), p53 (Abcam, ab240) and DNMT1 (Cell Signalling, D63A6). Images were revealed using the LI-COR infra-red imager using LI-COR infra-red secondary antibodies.

### Cell culture

All cell lines were grown in DMEM (Gibco, Waltham, MA, USA) supplemented with 10% Foetal bovine serum (FBS), L-glutamine (20 mM) and penicillin/streptomycin (50 ug/ml). The *TP53* null cell lines with stable ectopic expression of mutant p53^R175H^ were additionally supplemented with G418 (400 μg/ml). All cell lines were grown in a humidified cell culture incubator with 20% O_2_ and 5% CO_2_ at 37 °C.

### siRNA transfection

All siRNA transfections were achieved using Dharmafect 1 transfection reagent as directed by the manufacturer’s instruction. Both *DNMT1* (4390771) and control (4390844) siRNAs were purchased from the Silencer Select range of validated siRNAs (Thermo Fisher Scientific). A final concentration of 20 nM siRNA was used for all siRNA transfections. Both RNA and protein lysate were collected 48 h post transfection.

### 5-aza-Deoxycitidine treatment

Cells were treated with 1 nM 5-aza-deoxycitidine or an equivalent volume of vehicle (DMSO) for 48 h before being collected for analysis.

### Reverse Invasion assays

60 μl of Matrigel was placed into cell culture transwell inserts and incubated at 37 °C with 5% CO_2_ for 1 h in 12 well plates. The inserts were inverted and 100 μl of a 350,000 cells/ml suspension was applied to the membrane. Plates were incubated at 37 °C with 5% CO_2_, inverted, for 5 h to allow adherence. The plates were then reverted and the inserts were washed twice in serum free DMEM, before being placed into fresh wells containing 1 ml of serum free media. 100 μl of media with 10% serum and 10 ng/ml Hepatocyte Growth Factor (HGF) was then placed into the transwell and the cells were incubated at 37 °C and 5% CO_2_ for 48 h. Cells were stained in 4 nM Calciene for 1 h before being imaged using a confocal microscope.

Non-invasive cells were removed from the base of the membrane, before the transwell was placed onto a large coverslip on the stage of a confocal microscope. Z-stacks were taken from the inner surface of the membrane at intervals of 15 μm with a total of 15 images being taken. The same positions were used for each transwell in order to reduce user bias. Each experimental repeat consisted of 2 transwells per condition with 3 Z-stacks being taken per transwell.

Fluorescence for each image of a stack was quantified using ImageJ. A fluorescent signal of cells past 60 μm was considered invasive. The percentage of fluorescent signal past 60 μm was calculated as a percentage of the total fluorescent signal for each stack, and an average percentage of invasion was calculated for each transwell. Statistical analysis was carried out using a two-sample, two-tailed, paired *t*-test, comparing the average invasion of each transwell in a condition (*n* = 4). Conditions with overexpression of miR-142-3p included a preceding day, where cells were transfected with either an empty vector or one containing the miR-142 primary microRNA sequence. Transfected cells not used for the invasion assay were used to quantify the degree of miR-142-3p overexpression using Taqman assays.

### Analysis of human patient survival

The YM500v3 database was interrogated to see how *miR-142-3p* expression correlated with human patient survival. A single PDAC data set was used for the analysis. The ProGENEV2 database was used to investigate the effect of DNMT1 expression on human patient survival. Of the six PDAC data sets available, three (GSE28735, GSE50827 and GSE71729) had a *p* value of <0.1 and all correlations found that high *DNMT1* mRNA expression correlates with poor survival.

## Electronic supplementary material

Supplemental Figure 1

Supplementary figure legends
