# Supplementary material for: MiR-142-3p is downregulated in aggressive p53 mutant mouse models of pancreatic ductal adenocarcinoma by hypermethylation of its locus
Source: Cell Death Dis. 2018 May 29;9(6):644. doi: 10.1038/s41419-018-0628-4 (PMC5973943; doi:10.1038/s41419-018-0628-4)
Supplement: Supplementary file 1 — Supplemental Figure 1 [file 41419_2018_628_MOESM1_ESM.pdf]

Supplemental figure 1

Kras $p53^{flox}$ vs Kras $p53^{R172H}$

MicroRNA	FDR p-value	Fold change $p53^{flox}$ vs $p53^{R172H}$
mmu-miR-142-3p	0.015912686	4.579501
mmu-miR-378a-5p	0.015912686	100.90305
mmu-miR-142-5p	0.024677997	5.326641
mmu-miR-340-5p	0.03768438	2.1791608
mmu-miR-378b	0.04617887	1.9538167
mmu-miR-30c-2-3p	0.046220515	10.701015

Kras $Pten^{flox}$ vs Kras $p53^{R172H}$

MicroRNA	FDR p-value	Fold change $PTEN^{flox}$ vs $p53^{R172H}$
mmu-miR-99b-3p	7.69E-07	113.99959
mmu-miR-294-3p	2.79E-06	320.68503
mmu-miR-669b-5p	2.79E-06	419.04193
mmu-miR-669k-5p	2.79E-06	428.9504
mmu-miR-466j	2.94E-06	455.1476
mmu-miR-467c-5p	6.41E-06	246.54257
mmu-miR-669o-5p	8.97E-06	402.30725
mmu-miR-1897-3p	3.59E-05	723.20294
mmu-miR-669f-5p	3.92E-05	225.63422
mmu-miR-466c-5p	4.34E-05	190.55098
mmu-miR-21a-3p	9.33E-05	3.5364487
mmu-miR-30b-5p	4.35E-04	-3.4498224
mmu-miR-29a-5p	7.23E-04	-19.13431
mmu-miR-30c-5p	0.001151603	-5.138847
mmu-miR-466h-5p	0.001338091	235.14775
mmu-miR-466m-5p	0.002775021	292.85275
mmu-miR-574-3p	0.002775021	6.7297354
mmu-miR-5097	0.002927853	3.2289858
mmu-miR-669e-5p	0.004637764	201.83232
mmu-miR-30a-5p	0.007297209	-2.5347247
mmu-miR-877-3p	0.009261131	382.51346
mmu-miR-148a-3p	0.01450366	-6.1242614
mmu-miR-30a-3p	0.01450366	-4.0922456
mmu-miR-30e-5p	0.01450366	-2.3169453
mmu-miR-142-3p	0.015912686	-3.7405517
mmu-miR-5131	0.015912686	62.88183
mmu-miR-212-3p	0.018766908	2.346933
mmu-miR-2861	0.019531315	3.3543012
mmu-miR-1949	0.019827234	5.1397347
mmu-miR-669n	0.019827234	7.853199
mmu-miR-3474	0.021911005	156.62888
mmu-miR-206-3p	0.025069715	94.05248
mmu-miR-30d-5p	0.026969474	-2.074138
mmu-miR-700-3p	0.026969474	14.4577265
mmu-miR-146b-5p	0.02814093	-3.7359202
mmu-miR-26a-5p	0.02814093	-2.2823944
mmu-miR-680	0.02814093	3.9772446
mmu-miR-1896	0.029733874	48.88707
mmu-miR-466f-5p	0.029733874	38.74589
mmu-miR-468-3p	0.029733874	46.62059
mmu-miR-705	0.031001119	2.9528363
mmu-miR-29c-3p	0.03166246	-2.694654
mmu-miR-340-5p	0.03768438	-1.9938585
mmu-miR-669l-5p	0.038636	64.641045
mmu-miR-101c	0.04444342	-3.5635889
mmu-miR-141-3p	0.044611663	-14.1373825
mmu-miR-3096b-3p	0.04617887	3.1702912
mmu-miR-378b	0.04617887	-2.1360645
mmu-let-7a-1-3p	0.046220515	-21.262583
mmu-miR-1839-5p	0.046220515	-1.8479085
mmu-miR-188-5p	0.046220515	5.736661
mmu-miR-193a-3p	0.046220515	2.4403427
mmu-miR-26b-5p	0.046220515	-1.892828
mmu-miR-3077-5p	0.046220515	47.68718
mmu-miR-3082-5p	0.046220515	5.310294
mmu-miR-30c-2-3p	0.046220515	-15.577352
mmu-miR-32-3p	0.046220515	35.45265
mmu-miR-34a-5p	0.046220515	-4.537201
mmu-miR-500-3p	0.046220515	1.9526352
mmu-miR-29c-5p	0.04778983	-74.125465
mmu-miR-296-5p	0.049537104	2.870229
