# Supplementary material for: MiR-142-3p is downregulated in aggressive p53 mutant mouse models of pancreatic ductal adenocarcinoma by hypermethylation of its locus
Source: Cell Death Dis. 2018 May 29;9(6):644. doi: 10.1038/s41419-018-0628-4 (PMC5973943; doi:10.1038/s41419-018-0628-4)
Supplement: Supplementary file 2 — Supplementary figure legends [file 41419_2018_628_MOESM2_ESM.docx]

**Supplemental figure 1: List of all statistically significant microRNAs with associated fold changes.** Agilent microRNA microarrays were used to investigate the expression profiles of microRNAs from pancreatic tumour tissues from mice with the following genotypes: Kras Pten^flox^ (n=4), Kras p53^flox^ (n=5) and Kras p53^R172H^ (n=5). An ANOVA was used to assess statistical significance with Tukey’s HSD and a Benjamini-Hochberg moderated FDR p-value using 0.05 as a cut off.
